# Supplementary material for: Molecular Characterization of Heat-Induced HSP11.0 and Master-Regulator HSF from Cotesia chilonis and Their Consistent Response to Heat Stress
Source: Insects. 2021 Apr 4;12(4):322. doi: 10.3390/insects12040322 (PMC8066536; doi:10.3390/insects12040322)
Supplement: Supplementary file 1 [file insects-12-00322-s001.zip › supplementary files/Table S1..docx]

**Table S1.** Accession numbers of species compared in this study

| Species | Accession No. |
| --- | --- |
| HSP |  |
| *Lissorhoptrus oryzophilus* | AHE77382 |
| *Macrocentrus cingulum* | ACF21815 |
| *Pteromalus puparum* | ACO57620 |
| *Bemisia tabaci* | CED78718 |
| *Sarcophaga crassipalpis* | ABL06942 |
| *Spodoptera frugiperda* | QGA73374 |
| *Bombyx mori* | NP_001091794 |
| *Ooceraea biroi* | EZA56819 |
| *Ericerus pela* | AGE92594 |
| *Musca domestica* | AQY54363 |
| *Acyrthosiphon pisum* | ADP68029 |
| *Chilo suppressalis* | KY701308 |
| *Dinoponera quadriceps* | XP_014478994.1 |
| *Bombus impatiens* | XP_003491863.1 |
| *Ceratina calcarata* | XP_017876915.1 |
| *Nasonia vitripennis* | XP_001599992.1 |
| *Apis dorsata* | XP_006622783.1 |
| *Megachile rotundata* | XP_012154158.1 |
| HSF |  |
| *Bombyx mori* | AB623244 |
| *Apis mellifera* | XP_395321 |
| *Mamestra brassicae* | BAG07219 |
| *Helicoverpa armigera* | MG696150 |
| *Microplitis demolitor* | XP_014295710.1 |
| *Chelonus insularis* | XP_034948044.1 |
| *Diachasma alloeum* | XP_015126911.1 |
| *Fopius arisanus* | XP_011306046.1 |
| *Habropoda laboriosa* | KOC70601.1 |
| *Apis cerana* | XP_028520248.1 |
| *Apis dorsata* | XP_031367980.1 |
| *Drosophila melanogaster* | NP_476575.1 |
| *Drosophila simulans* | XP_016028338.1 |
| *Aphis gossypii* | XP_027847816.1 |
| *Diuraphis noxia* | XP_015372325.1 |
| *Galleria mellonella* | XP_026759219.1 |
| *Bicyclus anynana* | XP_023943343.1 |
| *Bos taurus* | AAI23712.1 |
| *Gallus gallus* | AFP54343.1 |
| *Capra hircus* | AFN69446.1 |
| *Carassius auratus* | AFM77715.1 |
| *Manduca sexta* | XP_030039220.1 |
| *Homo sapiens* | BAA13433.1 |
