# Supplementary figures and images for: Molecular Characterization of Heat-Induced HSP11.0 and Master-Regulator HSF from Cotesia chilonis and Their Consistent Response to Heat Stress
Source: Insects. 2021 Apr 4;12(4):322. doi: 10.3390/insects12040322 (PMC8066536; doi:10.3390/insects12040322)

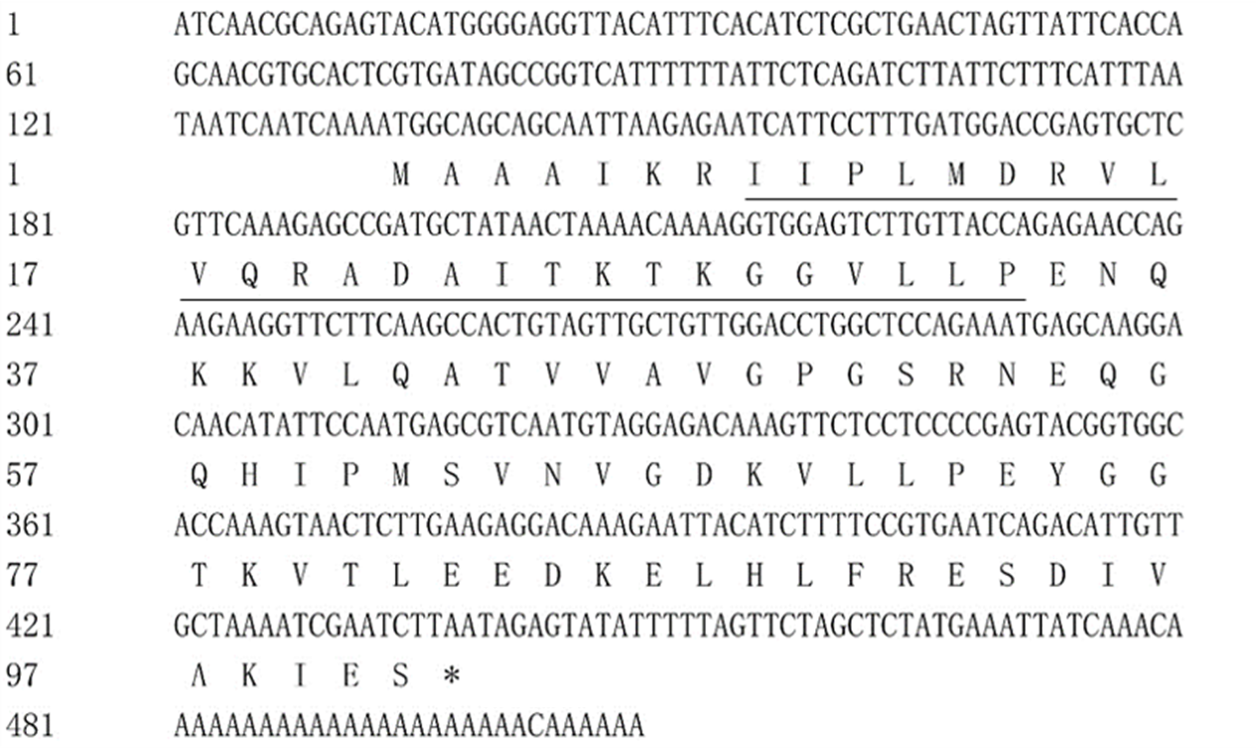

Supplement: Supplementary file 1 [file insects-12-00322-s001.zip › supplementary files/Fig. S1.png]

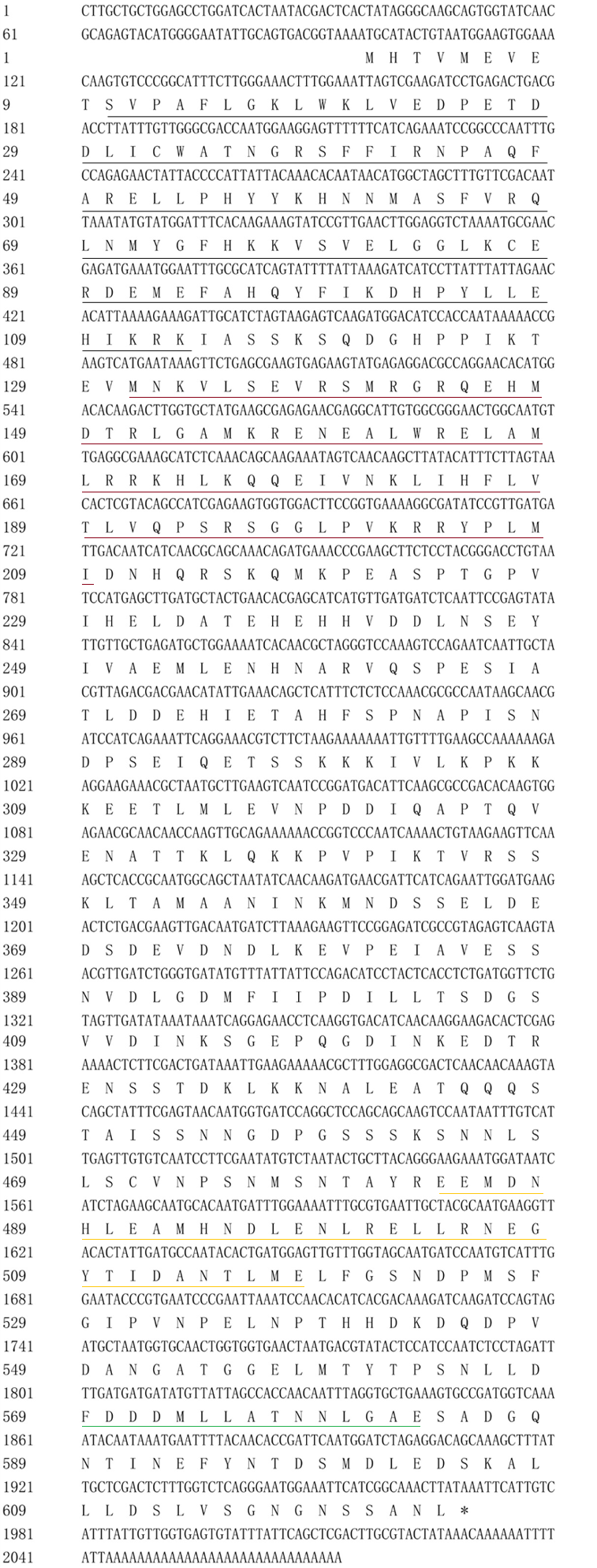

Supplement: Supplementary file 1 [file insects-12-00322-s001.zip › supplementary files/Fig. S2.png]
